# Quantitative Assessment of Anterior Segment OCT Parameters and Their Association with Intraocular Pressure in Primary Angle-Closure Disease

**DOI:** 10.3390/diagnostics16162496

**Published:** 2026-08-07

**Authors:** Alina-Gabriela Gheorghe, Tudor-George Potop, Dana-Margareta-Cornelia Dascalescu, Maria-Cristina Marinescu, Christiana-Diana-Maria Dragosloveanu, Ana-Maria Arghirescu, Ioana-Maria Rizea, Vasile Potop

**Affiliations:** 1 Department of Ophthalmology, Carol Davila University of Medicine and Pharmacy, 020021 Bucharest, Romania; 2 Department of Ophthalmology, Clinical Institute of Ophthalmological Emergencies “Prof. Dr. Mircea Olteanu”, 010464 Bucharest, Romania; 3 Medical Physiology Discipline, Carol Davila University of Medicine and Pharmacy, 020021 Bucharest, Romania; 4 Doctoral School, Carol Davila University of Medicine and Pharmacy, 020021 Bucharest, Romania

**Keywords:** AS-OCT, PACS, PAC, PACG, glaucoma, intraocular pressure (IOP), trabecular-iris space area (TISA), angle opening distance (AOD), cell density (CD), retinal nerve fiber layer (RNFL)

## Abstract

**Background/Objectives**: Glaucoma represents a progressive optic neuropathy and is the leading cause of irreversible blindness worldwide. The disease is classified as primary angle-closure glaucoma (PACG) or as primary open-angle glaucoma (POAG) depending on the iridocorneal angle. Although gonioscopy remains the gold standard investigation, anterior segment optical coherence tomography (AS-OCT) may provide important data about these structures, offering a quantitative assessment of the angle. The purpose of this study is to evaluate AS-OCT parameters in patients with primary angle-closure disease (PACD). **Methods**: This retrospective exploratory study included 75 eyes from 60 patients with PACD, classified as primary-angle closure suspect (PACS), primary-angle closure (PAC) and PACG. Participants underwent comprehensive ophthalmic examination: best-corrected visual acuity (BCVA), gonioscopy, AS-OCT, optic nerve OCT, visual field examination, and specular microscopy. Associations between AS-OCT angle parameters, intraocular pressure (IOP), and ocular biometrics were assessed using correlation analyses with Benjamini–Hochberg correction for the primary analyses and generalized estimating equation (GEE) models to account for inter-eye correlation. **Results**: In the overall PACD cohort, AS-OCT angle parameters were inversely associated with IOP, with all primary correlations remaining significant after multiple-testing correction. GEE analysis confirmed significant associations for TISA500, AOD500, and AOD750. Exploratory analyses in the PACS, PAC and PACG subgroups were not significant after correction for multiple testing, while anterior segment biometric parameters showed expected intercorrelations. **Conclusions**: Quantitative angle parameters demonstrated robust inverse associations with IOP in the overall PACD cohort. These exploratory findings support the complementary role of AS-OCT in the PACD anatomical evaluation and warrant confirmation in larger prospective studies to determine their clinical and prognostic relevance.

## 1. Introduction

Glaucoma represents a progressive optic neuropathy known as the leading cause of irreversible blindness in the world. The disease affects the retinal ganglion cells and optic nerve, resulting in corresponding changes in the visual field, with significant impact on the quality of life and activities of daily living [1]. The disease is divided based on the morphology of the iridocorneal angle, an anatomical structure located at the periphery of the anterior chamber (AC), between the iris and the cornea. In the iridocorneal angle, the structure of the trabecular meshwork can be found, which is the main aqueous humor (AH) outflow pathway of the eye, into the Schlemm canal (a circular structure located deep in the sclera) and further into the collector channels and episcleral veins [2]. As such, the disease is classified based on the mechanism of outflow obstruction. Primary angle-closure glaucoma (PACG) occurs when circumferential contact between the iris and trabecular meshwork (iridotrabecular contact, or ITC) physically blocks outflow. In contrast, primary open-angle glaucoma (POAG) involves structural accumulation of extracellular matrix within the trabecular meshwork [3].

Despite the fact that the prevalence of POAG is higher, PACG patients usually have more advanced optic neuropathy at presentation and a faster rate of disease progression [4]. The risk of blindness is known to be about 10% in POAG and 25% in PACG [3,4]. Considering that the optic nerve damage is permanent, understanding the mechanism of the disease is vital in order to detect glaucoma in early stages [5].

According to European Glaucoma Society (EGS) guidelines, primary angle-closure disease is categorized into three stages, all requiring ≥180° of iridotrabecular contact:

- Primary angle-closure suspect (PACS): normal IOP, absence of peripheral anterior synechiae (PAS), and no optic neuropathy;
- Primary angle-closure (PAC): elevated IOP and/or PAS, but no optic neuropathy;
- Primary angle-closure glaucoma (PACG): elevated IOP accompanied by glaucomatous optic neuropathy [4].

Other important morphological associations in angle-closure are: refractive error of hyperopia, short axial length (AL), shallow AC, high lens vault (LV) and lens thickness (LT) [1,4,6].

The PACG mechanism usually begins with appositional iridotrabecular contact that progresses to synechial iridotrabecular contact within the evolution of the disease. Two main overlapping mechanisms drive primary angle-closure (PAC): pupillary block—resistance to aqueous humor (AH) flow between chambers creates a pressure gradient that pushes the iris anteriorly into iridotrabecular contact; and Plateau iris configuration—anteriorly placed ciliary processes physically push the iris forward. Currently the degree of iridotrabecular contact and peripheral anterior synechia needed to affect the trabecular outflow and raise the IOP has not been identified [4].

Gonioscopy is still considered the reference clinical method for anterior chamber angle assessment. Through direct visualization of the angle, it provides essential information for the classification of glaucoma subtypes. Its usefulness in routine practice is reinforced by its accessibility, low cost, and rapid performance, requiring only a gonioscopy lens and a slit lamp. Nevertheless, the findings remain examiner-dependent and may be affected by the examination technique, the clinician’s level of experience, and patient cooperation [7].

In order to surpass these inherent limitations, several paraclinical methods have been devised for the investigation of the anterior chamber and support the diagnosis of angle-closure disease. Anterior segment Optic coherence tomography (AS-OCT) is able to provide important data about anterior chamber structures and offer a quantitative assessment of the AC angle configuration [8]. Studies have shown that AS-OCT can detect PAS almost as well as gonioscopy when performed in dark room conditions. Parameters such as trabecular iris space area (TISA) and anterior opening distance (AOD), along with LV, LT, and anterior chamber depth (ACD), have proven to be reliable in eyes with primary angle-closure suspect (PACS) and PACG [9].

We raise the hypothesis that quantitative AS-OCT metrics—specifically TISA and AOD—correlate with intraocular pressure and differ significantly across stages of primary angle-closure disease (PACD), reflecting progressive mechanical and functional trabecular outflow impairment. To test this hypothesis, the main objective of this study was to compare AS-OCT parameters (TISA 500, TISA 750, AOD 500, AOD 750, ACD, LV, LT) across patients with primary angle-closure suspect (PACS), primary angle-closure (PAC), and primary angle-closure glaucoma (PACG). Furthermore, we aim to identify correlations between TISA 500, TISA 750, AOD 500, AOD 750 and IOP (**primary analyses**) across disease stages and evaluate the relationship between anterior chamber parameters, central corneal thickness (CCT), and corneal endothelial cell density (CD) (**secondary analyses**). Accordingly, the contribution of this study is not the introduction of a novel AS-OCT metric or a diagnostic threshold, but an integrated, stage-stratified characterization of angle geometry and its cross-sectional associations with IOP, lens configuration and corneal endothelial cell density across the PACD spectrum.

## 2. Materials and Methods

We conducted a retrospective study, evaluating the data of patients who presented to the Clinical Institute of Ophthalmological Emergencies, “Prof. Dr. Mircea Olteanu”, Bucharest, Romania, between 1 January 2024 and 1 June 2026. The study follows the Declaration of Helsinki and was approved by the Ethics Committee of the Clinical Institute of Ophthalmological Emergencies, “Prof. Dr. Mircea Olteanu” (2171/25 May 2026).

All eyes were submitted to a complete ophthalmologic examination, including best-corrected distance visual acuity (BCVA), using a Snellen chart, Goldmann applanation tonometry, indentation gonioscopy, AS-OCT, optic nerve head (ONH) optical coherence tomography (OCT), specular microscopy for endothelial cell density measurement (CD) and visual field examination. IOP measurements were taken by the same trained physician using Goldmann applanation tonometry. Two measurements were taken and the mean value was used in the statistical analysis. All gonioscopic (carried out with a Zeiss gonio lens, Zeiss, Oberkochen, Germany) and AS-OCT (using the ANTERION OCT, Heidelberg Engineering, Heidelberg, Germany) measurements were performed under scotopic conditions by the same trained physician, in the same dark room, ensuring that the ambient illumination level was identical for all patients included in the study. The patient’s gaze was controlled using the device’s internal coaxial light fixation target. Subjects were given instructions to comfortably keep their chin and forehead firmly against the headrest and to maintain a steady and continuous focus on the central target. Proper centering was achieved between the corneal apex and the pupillary axis, as monitored in real time by the in-software pupil-tracking interface. For each eye, two AS-OCT scans were performed by the same trained physician, averaging the two readings for statistical analysis. Regarding quality control, the AS-OCT ANTERION Heidelberg system used in our study offers an integrated real-time quality indicator automatically classifying scans as “Pass,” “Borderline,” or “Fail.” To ensure the rigorous quality of the data, only scans labeled as “Pass” by the system for all three acquisition quality criteria (motion, fixation, tear film, and lid) and those that came out to be free of movement artifacts, eyelash or eyelid shadowing and misalignment relative to the center of the pupil or the apex of the cornea during the additional manual check were included in the final analysis. A total of 6 scans needed to be repeated, 4 of them because of “Borderline” results and 2 due to artifacts.

Scleral spur (SS) was identified by using a built-in algorithm for automatic detection of reference points in the ANTERION Metrics software. For accuracy, all automatically detected SS landmarks were independently and meticulously checked by two expert evaluators, in order to manually adjust the SS marker using the incorporated ruler editing tool if the system’s automatic detection was inaccurate. Automatic SS position was approved if the detected point correctly matched the anatomical apex of the internal scleral ridges along the corneoscleral junction. Manual adjustments were not necessary, since all automatically detected SS positions proved to be anatomically accurate within the imaging dataset included in the study.

In order to avoid diurnal IOP fluctuations, all patients were evaluated at the same time of the day, respectively between 8 and 10 a.m. Furthermore, to ensure that no anterior chamber geometry alteration occurred, we followed a strict protocol that required AS-OCT imaging to be performed prior to any contact procedures, as well as prior to pupillary dilation. Thus, the AS-OCT was performed first, followed by gonioscopy, followed by the IOP determination, in order to minimize the transient alterations of the angle that could appear during these clinical measurements, which would interfere with the OCT reading of the anterior chamber parameters. All the clinical and imaging exams were undertaken during the same clinical visit, with a maximum interval of 10 min between anterior segment scanning and IOP measurement.

Inclusion criteria consisted of angle closure on gonioscopy, defined as iridotrabecular contact for ≥180 degrees of the angle circumference. Exclusion criteria consisted of open angle on gonioscopy (scleral spur or ciliary body visible), any kind of secondary glaucoma (secondary mechanisms of angle closure, e.g., uveitis, neovascularization at the iridocorneal angle, tumors), if the patient was pregnant or breastfeeding, and any of the following significant ocular pathologies that could have compromised the image quality obtained by AS-OCT or visualization during gonioscopy, or those that could have made it difficult for patients to cooperate and thus led to erroneous measurements: corneal pathologies (e.g., leukoma-type corneal opacities, dystrophies, or pterygium); previous intraocular surgery (including aphakia and pseudophakia); incisional glaucoma surgery; refractive surgery; or concomitant pathologies of the posterior segment (e.g., diabetic retinopathy, age-related macular degeneration, retinal detachment, retinal dystrophies, or retinal vascular occlusions).

After applying the inclusion and exclusion criteria we divided the iridotrabecular contact patients into three groups: primary angle-closure suspect (PACS): normal IOP, no PAS, normal visual field and ONH OCT; primary angle-closure (PAC): IOP > 21 mmHg and/or PAS, normal visual field and ONH OCT; and primary angle-closure glaucoma (PACG): retinal nerve fiber layer (RNFL) loss and/or visual field changes.

The data analyzed in the present study were provided by the Humphrey visual field analyzer (mean deviation—MD), optic nerve OCT (cup-to-disc ratio—C/D), and AS-OCT. The technology employed by the AS-OCT system is based on Swept-Source OCT and a long wavelength of 1300 nm that provides high-resolution (<30 laterally × 10 axially μm) images. This device features a radial scanning design providing a 360° analysis, covered by 6 B-scans, with each one including 768 A-scans [10].

We determined several anterior chamber parameters: angle opening distance (AOD 500, AOD 750), defined as the distance between the iris and a 500 µm or 750 µm point anterior to the scleral spur (SS); and trabecular iris space area (TISA 500, TISA 750), defined as the surface limited by the anterior iris area, internal corneoscleral wall, a line that connects the iris and the SS, and AOD 500 and AOD 750, respectively [11]. Additional metrics were lens vault (LV), defined as the straight line connecting the anterior pole of the lens with the horizontal line linking the two scleral spurs; lens thickness (LT), defined as the distance between the anterior and posterior lens capsules; central corneal thickness (CCT), measured from the central point of the corneal epithelium to the central point of the corneal endothelium; anterior chamber depth (ACD), measured as the distance from the corneal endothelium to the anterior lens capsule; and anteroposterior axial length of the eye (AL), determined between the epithelial surface of the corneal apex and the retina [12,13,14,15].

### Statistical Analysis

Statistical analysis of the data was performed using the statistical package IBM SPSS Statistics for Windows, version 26 (IBM Corp., Armonk, NY, USA). This study includes both categorical and numerical, continuous data. The absolute and relative frequencies were calculated for categorical data. For numerical data, the average and standard deviation (SD) were determined. The normal distribution of the numeric variables was verified and confirmed using the Shapiro–Wilk Test—if significant (*p* value under 0.05), data significantly deviates from a normal distribution.

To evaluate differences across the three groups (PACS, PAC, PACG), we applied one-way analysis of variance (ANOVA) with post hoc Tukey HSD testing for normally distributed variables. For non-normally distributed variables, we used the Kruskal–Wallis test followed by post hoc Dunn testing with Bonferroni correction for multiple comparisons.

The degree of correlation between variables was ascertained by computing Spearman’s correlation coefficient, known as “Spearman’s rho”—as correlations are performed between variables with both normal and not normal distribution. A moderate correlation is between 0.3 and 0.5 or between −0.3 and −0.5, a strong correlation is over 0.5 or below −0.5, and a weak correlation is between 0.3 and −0.3 according to Spearman’s rho. To account for the increased risk of false-positive findings resulting from multiple correlation analyses, a Benjamini–Hochberg correction was applied to the 16 prespecified tests evaluating the associations of IOP with TISA500, TISA750, AOD500, and AOD750 in the overall cohort and in the PACS, PAC, and PACG subgroups (**primary analyses**). The false discovery rate was set at 5%, and Benjamini–Hochberg-adjusted *p*-values, reported as q-values, were considered statistically significant when they were below 0.05. Both the original unadjusted *p*-values and the corresponding q-values were considered when interpreting the results.

Because both eyes of some participants were included, the associations between anterior chamber angle parameters and IOP were re-evaluated using generalized estimating equations to account for the correlation between fellow eyes. Patient identity was specified as the clustering variable, and IOP was modeled using a Gaussian distribution with an identity link and robust sandwich standard errors. Separate models were fitted for TISA500, TISA750, AOD500, and AOD750 (**primary analyses**). In addition, two exploratory models included the corresponding TISA and AOD measurements simultaneously at 500 µm and 750 µm. These paired models were interpreted cautiously because TISA and AOD describe closely related aspects of angle anatomy and were strongly correlated.

A post hoc sensitivity analysis was performed using GPower version 3.1.9.7 to determine the minimum correlation magnitude that could be detected with the available sample sizes. The analysis was based on a two-sided bivariate correlation model, with a significance level of 0.05, a target statistical power of 80%, and a null correlation of zero. Separate calculations were performed for the associations of IOP with TISA500, TISA750, AOD500, and AOD750 in the overall cohort and in the PACS, PAC, and PACG subgroups. Because the primary analyses used Spearman’s rank correlation, the bivariate correlation model implemented in GPower was used as an approximation [16].

## 3. Results

### 3.1. Descriptive Results

**Figure 1:**
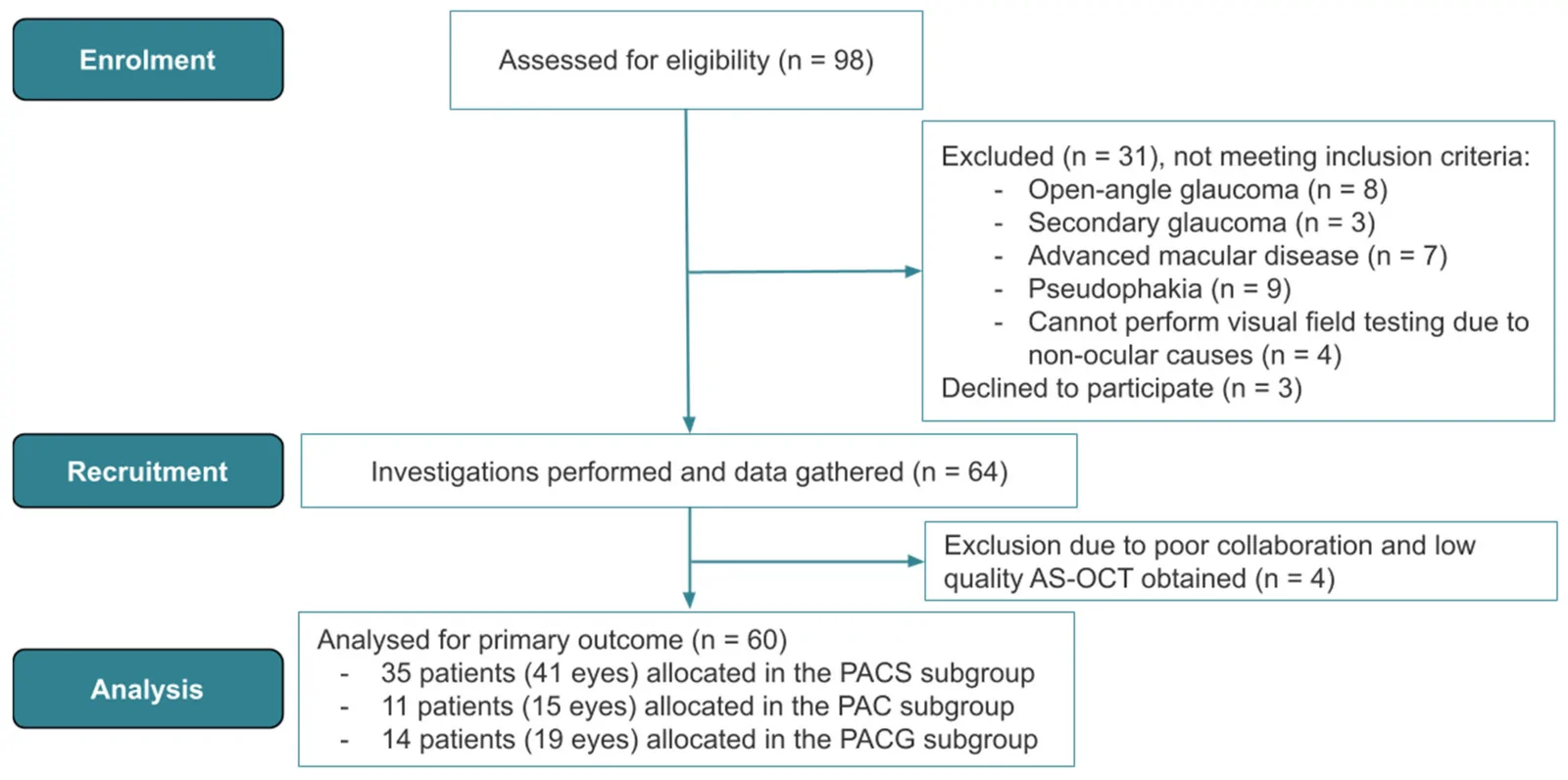
Flowchart showing patient selection, exclusions, and final cohort composition.

We conducted a study that included a total of 75 eyes from 60 patients: 41 eyes diagnosed as primary angle-closure suspects (PACS, 54.67%), 15 eyes diagnosed with primary angle-closure (PAC, 20%) and 19 eyes with primary angle-closure glaucoma (PACG, 25.33%). A patient enrollment flowchart can be found in Figure 1.

Average values of parameters followed in the study can be found in Table 1. Variables which are normally distributed are lens vault and thickness, while all other variables did not present normal distribution.

### 3.2. Significant Differences Between Subgroups

**Figure 2:**
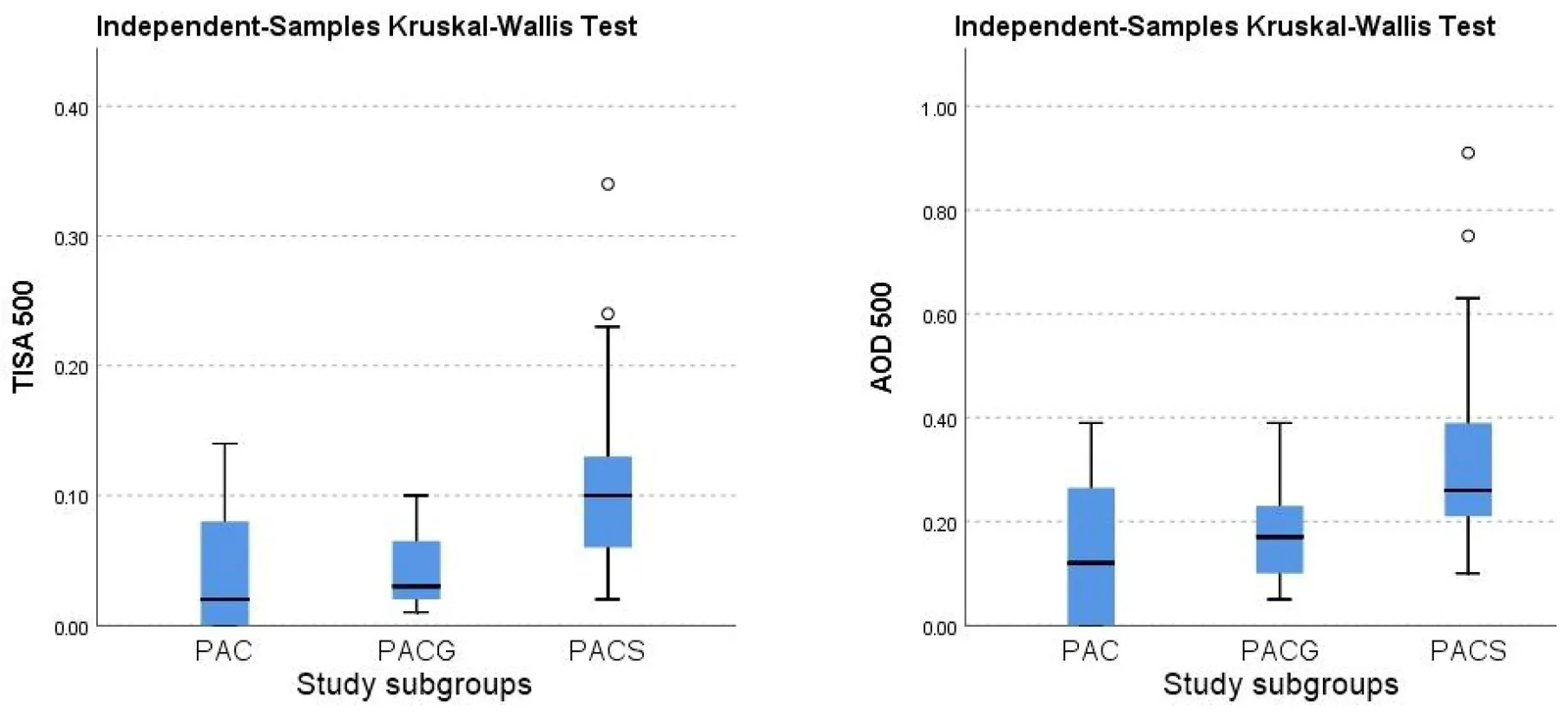
Boxplots displaying the interquartile range (blue rectangle), median value (horizontal line in the blue rectangle), minimum and maximum values, outlier values (circle) in the cohort of PAC, PACG and PACS regarding TISA 500 (first image) and AOD 500 (second image).

**Figure 3:**
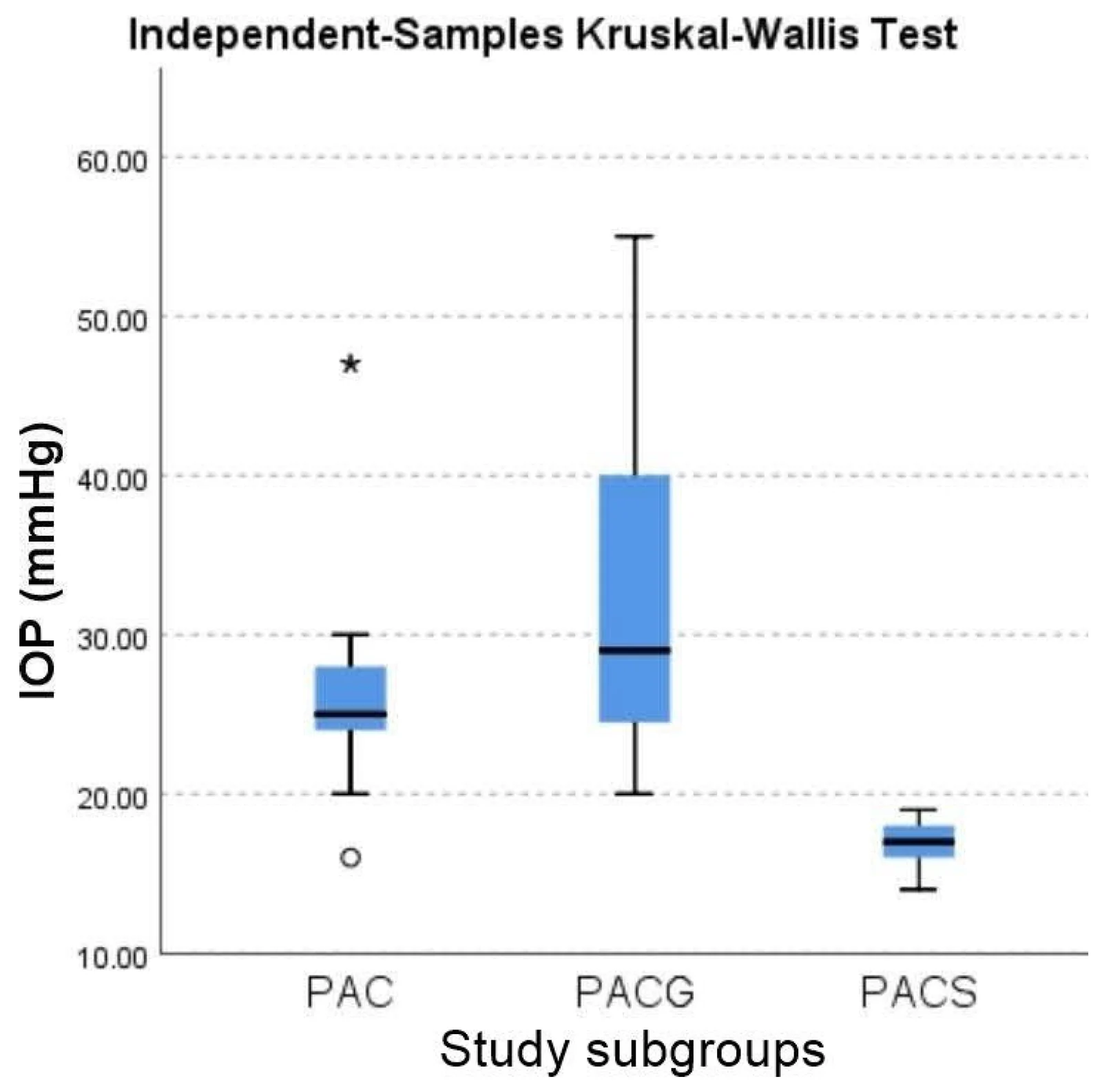
Boxplots displaying the interquartile range (blue rectangle), median value (horizontal line in the blue rectangle), minimum and maximum values, outlier values (circle) in the cohort of PAC, PACG and PACS regarding intraocular pressure (* *p* < 0.05).

Overall differences across the three subgroups were statistically significant for non-normally distributed variables, as determined by the Kruskal–Wallis test (Table 2, Figure 2 and Figure 3): TISA 500 (*p* < 0.001), TISA 750 (*p* = 0.001), AOD 500 (*p* < 0.001), AOD 750 (*p* = 0.008), IOP (*p* < 0.001), CD (*p* = 0.001), ACD (*p* = 0.002), C/D ratio (*p* < 0.001), MD (*p* = 0.004), and CCT (*p* = 0.042). No statistically significant differences were observed among subgroups for normally distributed variables, namely lens vault (*p* = 0.114) and lens thickness (*p* = 0.790).

Pairwise post hoc comparisons using Dunn’s test identified specific subgroup differences (Table 3):

- PACS vs. PAC: Significant differences were found for TISA 500 (*p* < 0.001), TISA 750 (*p* = 0.001), AOD 500 (*p* = 0.001), AOD 750 (*p* = 0.033), IOP (*p* < 0.001), CD (*p* = 0.003), and ACD (*p* = 0.004).
- PACS vs. PACG: Significant differences were observed for TISA 500 (*p* = 0.001), AOD 500 (*p* = 0.005), AOD 750 (*p* = 0.042), IOP (*p* < 0.001), C/D ratio (*p* < 0.001), and MD (*p* = 0.035).
- PAC vs. PACG: Significant differences were noted for C/D ratio (*p* < 0.001), MD (*p* = 0.015), and CCT (*p* = 0.036).

**Table 3:** Post hoc analysis showing which pair of subgroups presents statistically significant differences.

Variables	Pairs of Subgroups	*p* Value (Dunn Test)
TISA 500	PACS—PAC	<0.001
	PACS—PACG	0.001
TISA 750	PACS—PAC	0.001
AOD 500	PACS—PAC	0.001
	PACS—PACG	0.005
AOD 750	PACS—PAC	0.033
	PACS—PACG	0.042
IOP	PACS—PAC	<0.001
	PACS—PACG	<0.001
CD	PACS—PAC	0.003
ACD	PACS—PAC	0.004
C/D	PACS—PACG	<0.001
	PAC—PACG	<0.001
MD	PAC—PACG	0.015
	PACS—PACG	0.035
CCT	PAC—PACG	0.036

PACS—PAC differences are shown in blue, PACS—PACG differences are shown in purple, PAC—PACG differences are shown in red.

### 3.3. Correlations in the PAC Disease (PACD) Patient Group (Entire Cohort)

Firstly, the entire primary angle-closure disease cohort was analyzed using Spearman’s correlations for not normally distributed variables (see Table 4 for all statistically significant correlations). All anterior chamber parameters presented moderate-strong positive correlations amongst them (TISA 500 and 750, AOD 500 and 750, ACD), and they presented negative correlations with LT and LV. IOP presented negative moderate-strong correlations with TISA, AOD and ACD, and positive moderate correlation with LV. CD presented weak-moderate positive correlations with TISA, AOD and ACD and weak negative correlation with IOP. After applying the Benjamini–Hochberg correction, all four correlations (IOP with TISA 500, TISA 750, AOD 500, and AOD 750) assessed in the overall cohort remained statistically significant, with adjusted *p*-values of q ≤ 0.004. This indicates that the associations between IOP and TISA500, TISA750, AOD500, and AOD750 in the complete study population were robust to correction for multiple testing.

The sensitivity analysis showed that the overall cohort had sufficient sensitivity to detect correlations of moderate magnitude. The minimum detectable correlation was ∣ρ∣ = 0.319 for TISA 500—IOP, ∣ρ∣ = 0.323 for TISA 750—IOP, ∣ρ∣ = 0.321 for AOD 500—IOP, and ∣ρ∣ = 0.323 for AOD 750—IOP. Therefore, all correlations detected between these four pairs of variables were sensitive enough in the entire cohort.

### 3.4. Correlations in the PACS Subgroup

In the PACS subgroup, TISA 500, TISA 750, AOD 500, AOD 750 and ACD present strong positive correlations and moderate-strong negative correlations with LV and LT (see Table 5). Importantly, IOP and TISA 500 present a negative moderate correlation. After applying the Benjamini–Hochberg correction, none of the correlations assessed (IOP with TISA 500, TISA 750, AOD 500, and AOD 750) in the PACS cohort remained statistically significant.

In the PACS subgroup, the minimum detectable correlation ranged from ∣ρ∣ = 0.426 to ∣ρ∣ = 0.431. Therefore, this subgroup was adequately sensitive mainly to moderate-to-large associations, and none of the four tested correlations remains significant.

### 3.5. Correlations in the PAC Subgroup

The PAC subgroup presented significant correlation of IOP, CD and CCT with the AC parameters (TISA and AOD): the shallower the AC, the higher the IOP, the lower the CD and the thicker the central cornea. Furthermore, there are negative correlations between IOP—CD, IOP—ACD, and CD—CCT and positive correlations between IOP—CCT and CD—ACD (see Table 6). After applying the Benjamini–Hochberg correction, none of the correlations assessed (IOP with TISA 500, TISA 750, AOD 500, and AOD 750) in the PAC cohort remained statistically significant.

The sensitivity analysis showed that the smaller PAC subgroup had sufficient sensitivity to detect only correlations of approximately ∣ρ∣ ≥ 0.669 with 80% power for all four parameters. Therefore, the statistically significant correlations between TISA 500—IOP (ρ of −0.556) and TISA 750—IOP (ρ of −0.539) were below the detectable effect size associated with 80% power.

### 3.6. Correlations in the PACG Subgroup

Finally, the PACG group presented several significant correlations: moderate-strong positive correlations between AC parameters (TISA, AOD, ACD, LT, LV); negative correlations between TISA 500 and both IOP and CD (see Table 7). After applying the Benjamini–Hochberg correction, none of the correlations assessed (IOP with TISA 500, TISA 750, AOD 500, and AOD 750) in the PACG cohort remained statistically significant.

The sensitivity analysis showed that the PACG subgroup could detect correlations of approximately ∣ρ∣ ≥ 0.605 for TISA 500—IOP; therefore, the TISA 500—IOP (ρ of −0.483) correlation was below the detectable effect size associated with 80% power.

### 3.7. Generalized Estimating Equations (GEEs) of Main Correlations Identified

After accounting for the correlation between fellow eyes, greater TISA 500, AOD 500, and AOD 750 values remained associated with lower IOP. A 0.1 mm^2^ increase in TISA 500 was associated with an estimated 7.09 mmHg lower IOP (B = −7.09; 95% CI, −10.53 to −3.65; *p* < 0.001). Similarly, a 0.1 mm increase in AOD 500 was associated with a 2.44 mmHg lower IOP (B = −2.44; 95% CI, −3.74 to −1.15; *p* < 0.001), while a 0.1 mm increase in AOD 750 was associated with a 1.11 mmHg lower IOP (B = −1.11; 95% CI, −1.73 to −0.49; *p* < 0.001). The association between TISA 750 and IOP was not statistically significant in the corresponding GEE model (B = −0.11 per 0.1 mm^2^; 95% CI, −3.79 to 3.57; *p* = 0.954) (Table 8).

In the exploratory model including TISA 500 and AOD 500 simultaneously, TISA 500 remained inversely associated with IOP (B = −7.28 per 0.1 mm^2^; 95% CI, −12.26 to −2.29; *p* = 0.004), whereas AOD 500 did not retain a significant association (*p* = 0.937). In the corresponding 750 µm model, AOD 750 remained associated with lower IOP (B = −1.80 per 0.1 mm; 95% CI, −3.49 to −0.12; *p* = 0.035), whereas TISA 750 was not significant (*p* = 0.363). Because of the strong correlation between the paired angle parameters, these mutually adjusted estimates were considered exploratory.

## 4. Discussion

To synthesize, our study reveals that, in this Romanian PACD cohort, several anatomical and functional parameters are significantly different between different stages of the disease, particularly between the incipient PACS and the PAC/PACG stages. These findings indicate that AS-OCT parameters vary according to disease stage and may provide complementary anatomical information when evaluating patients with primary angle-closure disease.

The **primary analysis** consisted of a correlation between AS-OCT angle parameters (TISA 500 and 750, AOD 500 and 750) and IOP, which were all statistically significant, negative and strong in the global PACD cohort. Moreover, these findings had reasonable sensitivity to identify moderate associations between anterior chamber angle parameters and IOP. The Benjamini–Hochberg correction retained significance in all four correlations, suggesting that the associations observed in the overall cohort are unlikely to be explained solely by the number of statistical tests performed. The GEE analysis was performed to address the non-independence introduced by the inclusion of both eyes from some participants. The significant inverse associations of TISA500, AOD500, and AOD750 with IOP were maintained after accounting for within-patient correlation, while the association involving TISA750 did not remain significant in the clustered analysis.

The **secondary, exploratory** analysis involved the correlation of TISA 500 and 750, AOD 500 and 750 and IOP in the three subgroups, of which the most consistently significant (in all three groups) was the IOP—TISA 500 correlation. However, the results were below the detectable effect size associated with 80% power and the correction for multiple tests rendered all associations non-significant.

Other **secondary, exploratory** analyses involved correlations between anterior chamber parameters (TISA, AOD but also LV, LT, ACD), endothelial cell density and intraocular pressure. Because these analyses were exploratory and were not included in the pre-specified multiple-testing correction, they should be interpreted as hypothesis-generating and require confirmation in independent cohorts. Most consistent (significant in the PACD, PACS, PAC and PACG groups) were TISA 500—AOD 500, TISA 500—AOD 750, TISA 750—AOD 500, TISA 750—AOD 750, AOD 500—AOD 750 and LV—LT, reinforcing the internal consistency of the measurements and reflecting the close anatomical relationship among anterior segment biometric parameters. Because these measurements describe related components of anterior segment anatomy, they should not be interpreted as independent variables, and the observed associations with IOP should be considered descriptive rather than indicative of independent predictive effects.

Our study is compatible with other studies that show the importance of AS-OCT parameters in evaluating angle-closure patients [17,18,19]. So far, there is no clear definition of angle-closure on AS-OCT, and gonioscopy remains the gold standard investigation in this regard. The procedure is inexpensive, fast and can be performed in almost any ophthalmology practice. However, it does not provide quantitative data and is dependent on the examiner, so it proves hard to duplicate [20].

The importance of the iridocorneal angle was highlighted by many studies, including the EGS guidelines. PAC disease consists of iridotrabecular contact of more than 180 degrees with or without high IOP and glaucomatous optic neuropathy [4]. Mou et al. [18] achieved promising results for PACD opportunistic screening using AC parameters measured with AS-OCT, with a 91.11% specificity. They reported significantly higher values for LT and LV, lower TISA 500 and AOD 500 and smaller ACD in PAC disease patients compared with normal subjects [18].

Lower values of TISA 500 and 750, AOD 500 and 750 and higher mean IOP in PAC and PACG subgroups are consistent with studies that show higher IOP and lower TISA 500 in eyes with PAS [21,22]. Also, PAC and PACG groups differ by MD, consistent with the natural evolution of the disease, which presents glaucomatous neuropathy in the PACG stage [23]. Interestingly, IOP did not differ significantly between PAC and PACG stages according to the disease progression. This may have important clinical implications, as the question of progression of PACS to PAC and PACG has been raised by several prospective studies, particularly the seminal ZAP study (Zhongshan Angle-Closure Prevention Trial) [24]. The AS-OCT investigation of the ZAP cohort identified AOD 500, AOD 750 and TISA 500 as significantly smaller in PACS eyes which progressed, compared to PACS eyes which did not progress, throughout the study follow-up—similar to our study, in which these parameters significantly differed between PACS and the more advanced PACD stages. Interestingly, LT did not differ between stages in our study and did not correlate with IOP, in contrast with the ZAP study, in which it was significantly higher in the progressor subgroup—suggesting that the lens volume and position contribution in angle closure requires more studies and may differ between different ethnic groups [24]. In an Indian population, the risk of progression in five years was reported to be 22% from PACS to PAC and 28.5% from PAC to PACG [25].

In line with our findings, Guzman et al. observed significant differences in TISA and AOD between PACS and PAC as well as between PACS and PACG. They also reported a lack of statistically significant differences in these parameters within the PAC versus PACG analysis [19]. While the inconsistent relationships reported in the present study may be due in part to the small cohort, a large study of almost 300 eyes also failed to find significant differences in AS-OCT parameters between PACS and PAC/PACG groups. However, the latter identified significant differences in sectorial measurements (superonasal and superotemporal TISA 500, inferonasal TISA 500 and TISA 750) while attempting the integration into a machine learning algorithm, suggesting that the utility of AS-OCT in differentiating PACD stages may lie in the integration of several distinct AC parameters [26].

The primary result of our study consists of robust correlation between IOP and AC parameters in the larger PACD group. Similar results have been obtained in a population-based epidemiological study (the Chinese American Eye Study), in which no IOP correlation exists in the open angle control group, but the correlations to TISA 500 and AOD 500 were similar to our study, while the correlation with TISA 750 was stronger and the correlation with AOD 750 was weaker compared to our rho coefficients, suggesting a role for subtle anatomical differences in different angle closure populations [21].

Regarding the secondary analysis, the lens parameters (vault, thickness) and the AC parameters (AOD, TISA, ACD) were variably correlated to each other in the PACD study cohort, as expected, as they reflect the biological process of anterior chamber shallowing and are strongly collinear variables. A multivariate analysis performed on 425 PACD eyes revealed that ACD, LV, AOD 750 and TISA 750 were significantly associated with acute angle closure, with ACD being the strongest predictor [19].

Another set of **secondary, exploratory** analyses involved correlations of endothelial cell density: there were significant, weak-moderate positive correlations between CD and TISA 500, TISA 750, AOD 500 and ACD in the PACD group; however, the correlations were very different between disease stages: in PACS, AOD 500—CD presented a moderate positive correlation, in PAC a moderate-strong positive correlation between CD and either TISA 500 or TISA 750 and AOD 500, while in PACG strong negative correlations were found between CD and either TISA 500, TISA 750, AOD 500, or AOD 750 and ACD. These internally consistent correlations may indicate a real phenomenon (not only due to the small number of eyes) in which glaucomatous eyes with advanced AC narrowing have an inverse correlation with cell density. Several pathophysiological mechanisms may be involved in these reversed correlations: firstly, in incipient PACD (PACS and PAC) the main driver of endothelial damage may be chronic intermittent iridocorneal contact, as it was demonstrated experimentally that aqueous humor convection in a shallow anterior chamber creates more shear stress to the corneal endothelium [27]. On the other hand, in the more advanced PACG, the endothelial count becomes independent strictly from the chamber depth, as more aggravating factors in endothelium survival intervene: previous acute angle closure episodes and higher overall IOP (higher cell loss in longer attack duration) and even PACG therapeutic interventions (laser peripheral iridoplasty and iridotomy) [28,29].

Such results have not been widely replicated previously, such as in similar cohorts to our study in which no correlation between CD and ACD was detected, across the PACD stages [30]. More extensive studies are needed to clarify the relationship between endothelial health and the angle-closure spectrum.

A detailed understanding of the anterior chamber morphology in PACD is essential in therapeutic planning, particularly surgical. IOP is known as the most important risk factor in glaucoma and the only one that can be influenced with treatment. Data demonstrates the importance of IOP in glaucoma progression and structural damage of the RNFL [31]. Surgically, maintaining IOP within target can be achieved with lens extraction, Minimally Invasive Glaucoma Surgery (MIGS) or filtration surgery [32]. Considering that PACG patients often present late, with irreversible RNFL loss, it is important to evaluate and treat each eye prior to the appearance of visual field changes [33,34,35]. EGS guidelines suggest that chronic angle-closure with high IOP (more than 35 mmHg) associated with PAS and glaucomatous optic neuropathy should be considered for glaucoma surgery in order to establish IOP control [4]. Not last, the utility of the peripheral laser iridotomy has been investigated in a significant longitudinal cohort in the ZAP study, revealing that the risk ratio of progression from PACS to PAC is 0.31 compared to control PACS eyes; however, no consistent predictors for progression have been accurately pinpointed yet [36].

As the study was retrospective and involved patients presenting to a tertiary center, cataract patients were included, in which lens opacification often involves volume increase and decreased BCVA and can determine a lower anterior chamber and higher LV, which may ultimately occlude the angle and raise the IOP. Although initially the disease can be asymptomatic, it may affect the trabecular meshwork and the aqueous humor filtration depending on the time spent with high IOP. Other studies have shown that lens extraction may achieve adequate IOP reduction when performed shortly after the onset of IOP elevation or following an episode of acute angle closure [37,38,39]. Furthermore, the EAGLE study revealed that early clear lens extraction can be a useful tool in PAC and PACG patients [32]. On the other hand, in cases of extensive PAS, adequate IOP control may not be achieved with lens extraction alone. Therefore, the management of angle-closure patients should be individualized, taking into account multiple factors, including the duration of IOP elevation before presentation, the extent of PAS, and the severity of optic nerve damage [3].

Studies have shown that AS-OCT plays an important role in the evaluation of patients with angle closure that have impaired AH outflow through the trabecular meshwork [40]. The advantage is that AS-OCT provides objective data that can be repeated, using the same software, with similar results regardless of the investigator [41]. AS-OCT also has some disadvantages, such as the fact that it cannot distinguish iridotrabecular contact and PAS [42]. In order to compensate for this limitation, Su et al. suggested a TISA 500 threshold value of 0.046 mm^2^ that symbolizes the level where PAS might be present and IOP will rise [9,32]. Once PAS are present and the IOP rises, IOP-lowering interventions are recommended, such as laser peripheral iridotomy or lens extraction. While it cannot replace gonioscopy, anterior chamber angle imaging such as AS-OCT may play a complementary role in diagnosis and treatment choice [42].

Future directions of our work will include the expansion of the study group, particularly the PAC and PACG subgroups, in order to achieve adequate power and significance in the subgroup analyses. An important characteristic in PAC and PACG is the potential for PAS, which were not assessed in our study; however, we wish to expand the cohorts in order to adequately compare eyes with and without PAS. Other studies have shown a 0.046 value of TISA 500 that associated PAS [9]. Also, as we perform gonioscopy and AS-OCT in dark conditions in order to find the smallest iridocorneal angle in each patient, we could also measure IOP under dark conditions for the similar purpose of identifying maximal IOP value [43]. Moreover, taking into account preexisting studies that centered corneal biomechanical properties in glaucoma and identified the impact of corneal hysteresis as an independent risk factor, we aim to investigate the relationship between the latter, CD and AS-OCT parameters in PACD [44,45]. While the current cohort could not support an ROC analysis, future larger cohorts might be powered enough to establish clinically useful thresholds of TISA and AOD in order to identify clinically significant trabecular dysfunction and identify cases which need early lens extraction.

Regarding **potential limitations**, the present study is limited by its retrospective nature, which does not allow following the evolution of the patient. Follow-up studies are planned in order to examine the treatment response to different surgical and interventional methods of angle widening. Furthermore, the current study does not allow for the determination of cut-off values to establish the diagnostic accuracy of these parameters, since the establishment of threshold values requires a strictly independent and blinded evaluation of the two methods—the AS-OCT test and the gold standard method—which, in our study protocol, were performed by the same trained evaluator. This will remain a subject for future studies involving larger patient cohorts. Another limitation of this study is that patients were evaluated without discontinuing their topical pressure-lowering treatment. This decision was made for ethical reasons to prevent the risk of optic nerve damage caused by uncontrolled intraocular pressure spikes, although this may influence the observed correlations.

Another limitation is that multivariable regression analyses were not performed, and several evaluated variables—including TISA, AOD, anterior chamber depth, lens vault, and lens thickness—represent closely related aspects of anterior segment anatomy and demonstrated substantial intercorrelation. Given the relatively small sample size, particularly within the PAC and PACG subgroups, multivariable modeling would be susceptible to multicollinearity and unstable regression estimates. Accordingly, the present findings should be interpreted as association-based rather than evidence of independent predictive effects. Regarding the post hoc sensitivity analysis, the overall cohort was sufficiently sensitive to detect moderate correlations and the subgroup analyses were adequately sensitive only to larger effects. Therefore, nonsignificant subgroup results may partly reflect limited statistical sensitivity and an increased risk of type II error rather than the true absence of an association. Finally, a notable limiting factor of this study is the relatively small sample size and the uneven distribution of the eyes across the PACD subgroups. Although our distribution reflects clinical presentation rates in real-world practice, the small sample size—particularly of the smallest category (PAC)—inevitably limits the statistical power of our comparisons. Consequently, this increases the risk of type II errors, meaning that any negative results reported in this study should be interpreted with caution, as there may be real but undetected morphological or clinical differences because of this limited cohort.

## 5. Conclusions

Angle assessments can be done by gonioscopy, which offers a 360-degree evaluation but is dependent on the clinician, or by a device which may be dependent on technique and software.

Management of each patient should be evaluated thoroughly in order to choose the best therapeutic option to maintain the IOP within normal limits and to prevent further damage of the RNFL. Further studies are needed to clarify and confirm AS-OCT can be used as a predictor for developing PACG.

AS-OCT parameters such as TISA 500, TISA 750, AOD 500 and AOD 750 may be helpful in differentiating patients with efficient trabecular meshwork filtration and those with nonfunctional trabecular filtration. Further studies evaluating IOP are needed to confirm whether AS-OCT parameters can guide therapeutic decisions. Nonetheless, AS-OCT parameters remain valuable clinical adjuncts alongside gonioscopy for evaluating the iridocorneal angle.

## Figures and Tables

**Table 1:** Median, mean values, standard deviation (SD), and distribution of the parameters studied in the PACS, PAC and PACG subgroups and in the entire cohort.

Variable	Study Group	Mean	SD	Median	Normal Distribution (Shapiro–Wilk *p* Value)
TISA 500 (mm^2^)	PACS	0.111	0.068	0.10	No (<0.001)
	PAC	0.044	0.052	0.02	
	PACG	0.045	0.028	0.03	
	Total	0.081	0.066	0.06	
TISA 750 (mm^2^)	PACS	0.201	0.112	0.18	No (<0.001)
	PAC	0.086	0.098	0.06	
	PACG	0.180	0.222	0.12	
	Total	0.172	0.148	0.13	
AOD 500 (mm)	PACS	0.317	0.178	0.26	No (<0.001)
	PAC	0.139	0.151	0.12	
	PACG	0.179	0.098	0.17	
	Total	0.246	0.173	0.23	
AOD 750 (mm)	PACS	0.428	0.248	0.35	No (<0.001)
	PAC	0.237	0.193	0.23	
	PACG	0.268	0.128	0.26	
	Total	0.352	0.230	0.32	
BCVA	PACS	0.215	0.107	0.20	No (<0.001)
	PAC	0.198	0.069	0.20	
	PACG	0.241	0.209	0.20	
	Total	0.218	0.135	0.20	
IOP (mmHg)	PACS	16.683	1.386	17.00	No (<0.001)
	PAC	27.533	8.790	25.00	
	PACG	33.895	11.020	30.00	
	Total	23.213	10.095	18.00	
CD (cells/mm^2^)	PACS	2554.600	286.604	2551.00	No (<0.001)
	PAC	2167.933	429.929	2261.00	
	PACG	2453.474	215.168	2467.00	
	Total	2450.257	336.296	2480.50	
ACD (mm)	PACS	2.561	0.466	2.53	No (0.040)
	PAC	2.145	0.366	2.16	
	PACG	2.282	0.425	2.26	
	Total	2.411	0.467	2.38	
LV (mm)	PACS	0.578	0.329	0.57	Yes (0.094)
	PAC	0.739	0.275	0.72	
	PACG	0.725	0.372	0.74	
	Total	0.648	0.335	0.65	
LT (mm)	PACS	4.698	0.437	4.67	Yes (0.533)
	PAC	4.713	0.395	4.56	
	PACG	4.676	0.418	4.65	
	Total	4.696	0.419	4.67	
C/D ratio	PACS	0.320	0.071	0.30	No (<0.001)
	PAC	0.400	0.136	0.40	
	PACG	0.888	0.116	0.95	
	Total	0.489	0.261	0.40	
MD (dB)	PACS	−5.310	0.760	−5.29	No (0.005)
	PAC	−4.872	1.750	−4.80	
	PACG	−17.856	8.860	−17.50	
	Total	−12.257	9.250	−7.10	
CCT (micrometers)	PACS	553.406	33.032	552.00	No (<0.001)
	PAC	594.769	78.816	581.00	
	PACG	528.364	38.247	548.00	
	Total	558.089	52.381	552.00	

**Table 2:** Kruskal–Wallis test results for variables which are not normally distributed—differences between three groups.

	TISA 500	TISA 750	AOD 500	AOD 750	IOP	CD	ACD	C/D	MD	CCT
Kruskal- Wallis H	22.33	13.70	17.61	9.76	52.52	14.03	12.09	34.94	11.17	6.32
*p* value	<0.001	0.001	<0.001	0.008	<0.001	0.001	0.002	<0.001	0.004	0.042

**Table 4:** Statistically significant correlations between variables in the entire study cohort. * Primary hypothesis tests (Benjamini–Hochberg correction was applied to these four pre-specified correlations).

Correlations	Spearman’s Rho (*p* Value)	Correlations	Spearman’s Rho (*p* Value)
TISA 500—IOP *	−0.628 (<0.001)	TISA 750—IOP *	−0.429 (<0.001)
TISA 500—AOD 500	0.881 (<0.001)	TISA 750—AOD 500	0.829 (<0.001)
TISA 500—AOD 750	0.792 (<0.001)	TISA 750—AOD 750	0.808 (<0.001)
TISA 500—LV	−0.510 (<0.001)	TISA 750—LV	−0.585 (<0.001)
TISA 500—ACD	0.596 (<0.001)	TISA 750—ACD	0.639 (<0.001)
TISA 500—CD	0.305 (0.008)	TISA 750—CD	0.272 (0.021)
TISA 500—TISA 750	0.873 (<0.001)	TISA 750—LT	−0.306 (0.009)
AOD 500—IOP *	−0.507 (<0.001)	AOD 750—IOP *	−0.386 (0.001)
AOD 500—LV	−0.600 (<0.001)	AOD 750—LV	−0.667 (<0.001)
AOD 500—LT	−0.246 (0.034)	AOD 750—LT	−0.372 (0.001)
AOD 500—ACD	0.717 (<0.001)	AOD 750—ACD	0.752 (<0.001)
AOD 500—CD	0.321 (0.006)	AOD 500—AOD 750	0.838 (<0.001)
ACD—CCT	−0.267 (0.047)	LV—IOP	0.304 (0.008)
ACD—LV	−0.754 (<0.001)	CD—IOP	−0.253 (0.029)
ACD—LT	−0.484 (<0.001)	**Correlations**	**Pearson’s r (*p* value)**
ACD—IOP	−0.413 (<0.001)		
ACD—CD	0.244 (0.039)	LV—LT	0.745 (<0.001)

**Table 5:** Statistically significant correlations between variables in the PACS group. * Primary hypothesis tests (Benjamini–Hochberg correction was applied to these four pre-specified correlations).

Correlations	Spearman’s Rho (*p* Value)	Correlations	Spearman’s Rho (*p* Value)
TISA 500—IOP *	−0.346 (0.027)	TISA 500—TISA 750	0.975 (<0.001)
TISA 500—AOD 500	0.819 (<0.001)	TISA 750—AOD 500	0.880 (<0.001)
TISA 500—AOD 750	0.871 (<0.001)	TISA 750—AOD 750	0.944 (<0.001)
TISA 500—LT	−0.373 (0.018)	TISA 750—LT	−0.465 (0.002)
TISA 500—LV	−0.572 (<0.001)	TISA 750—LV	−0.669 (<0.001)
TISA 500—ACD	0.581 (<0.001)	TISA 750—ACD	0.692 (<0.001)
AOD 500—LT	−0.492 (0.001)	AOD 750—LT	−0.619 (<0.001)
AOD 500—LV	−0.650 (<0.001)	AOD 750—LV	−0.778 (<0.001)
AOD 500—ACD	0.707 (<0.001)	AOD 750—ACD	0.812 (<0.001)
AOD 500—CD	0.387 (0.015)	AOD 500—AOD 750	0.902 (<0.001)
ACD—LT	−0.741 (<0.001)	**Correlations**	**Pearson’s r (*p* value)**
ACD—LV	−0.768 (<0.001)	LV—LT	0.806 (<0.001)

**Table 6:** Statistically significant correlations between variables in the PAC group. * Primary hypothesis tests (Benjamini–Hochberg correction was applied to these four pre-specified correlations).

Correlations	Spearman’s Rho (*p* Value)	Correlations	Spearman’s Rho (*p* Value)
TISA 500—IOP *	−0.556 (0.031)	TISA 750—IOP *	−0.539 (0.038)
TISA 500—AOD 500	0.966 (<0.001)	TISA 750—AOD 500	0.992 (<0.001)
TISA 500—AOD 750	0.603 (0.017)	TISA 750—AOD 750	0.649 (0.009)
TISA 500—CCT	−0.848 (<0.001)	TISA 750—CCT	−0.873 (<0.001)
TISA 500—CD	0.527 (0.044)	TISA 750—CD	0.561 (0.030)
AOD 500—CCT	−0.873 (<0.001)	TISA 500—TISA 750	0.983 (<0.001)
AOD 500—CD	0.604 (0.017)	IOP—ACD	−0.636 (0.011)
AOD 500—AOD 750	0.661 (0.007)	IOP—CD	−0.673 (0.006)
**Correlations**	**Pearson’s r (*p* value)**	IOP—CCT	0.707 (0.007)
		CD—CCT	−0.650 (0.016)
LV—LT	0.782 (0.001)	CD—ACD	0.821 (<0.001)

**Table 7:** Statistically significant correlations between variables in the PACG group. * Primary hypothesis tests (Benjamini–Hochberg correction was applied to these four pre-specified correlations).

Correlations	Spearman’s Rho (*p* Value)	Correlations	Spearman’s Rho (*p* Value)
TISA 500—IOP *	−0.483 (0.036)	TISA 500—TISA 750	0.636 (0.006)
TISA 500—AOD 500	0.864 (<0.001)	TISA 750—AOD 500	0.678 (0.003)
TISA 500—AOD 750	0.743 (0.001)	TISA 750—AOD 750	0.755 (<0.001)
TISA 500—CD	−0.666 (0.002)	TISA 750—CD	−0.533 (0.028)
AOD 500—CD	−0.838 (<0.001)	AOD 750—CD	−0.714 (0.001)
AOD 500—AOD 750	0.880 (<0.001)	**Correlations**	**Pearson’s r (*p* value)**
ACD—CD	−0.539 (0.026)	LV—LT	0.695 (0.001)

**Table 8:** Generalized estimating equation models evaluating the associations between anterior chamber angle parameters and intraocular pressure.

Robust GEE Coefficient Estimates												
Model	Specification	Predictor	Effect Unit	N Eyes	N Clusters	B	Robust SE	95% CI Lower	95% CI Upper	Wald z	*p*-Value	Significant
GEE—1	Univariable: TISA 500	TISA 500	per 0.1 mm^2^	75	56	−7.092	1.755	−10.532	−3.652	−4.041	0.0001	Yes
GEE—2	Univariable: TISA 750	TISA 750	per 0.1 mm^2^	73	54	−0.109	1.879	−3.791	3.574	−0.058	0.9539	No
GEE—3	Univariable: AOD 500	AOD 500	per 0.1 mm	75	56	−2.443	0.660	−3.735	−1.150	−3.703	0.0002	Yes
GEE—4	Univariable: AOD 750	AOD 750	per 0.1 mm	73	54	−1.111	0.317	−1.732	−0.489	−3.503	0.0005	Yes
GEE—5	Multivariable TISA 500 + AOD 500	TISA 500	per 0.1 mm^2^	75	56	−7.276	2.542	−12.259	−2.293	−2.862	0.0042	Yes
GEE—5	Multivariable: TISA 500 + AOD 500	AOD 500	per 0.1 mm	75	56	0.078	0.987	−1.856	2.012	0.079	0.9371	No
GEE—6	Multivariable: TISA 750 + AOD 750	TISA 750	per 0.1 mm^2^	73	54	1.687	1.855	−1.948	5.322	0.910	0.3630	No
GEE—6	Multivariable: TISA 750 + AOD 750	AOD 750	per 0.1 mm	73	54	−1.805	0.858	−3.487	−0.123	−2.103	0.0355	Yes

Separate GEE models were fitted for each angle parameter using patient identity as the clustering variable and robust sandwich standard errors. Effect estimates are presented per 0.1 mm^2^ increase for TISA measurements and per 0.1 mm increase for AOD measurements. The paired TISA-AOD models were exploratory because of the strong correlation between the corresponding anatomical parameters. Significant results in green.

## Data Availability

The original contributions presented in this study are included in the article. Further inquiries can be directed to the corresponding authors.

## Abbreviations

The following abbreviations are used in this manuscript:

AC	Anterior chamber
ACD	Anterior chamber depth
ACG	Angle-closure glaucoma
AH	Aqueous humour (AH)
AI	Artificial intelligence
AL	Axial length
ANOVA	One-way analysis of variance
AOD	Angle opening distance
AOD 500	Angle opening distance measured at 500 µm
AOD 750	Angle opening distance measured at 750 µm
AS-OCT	Anterior segment optical coherence tomography
BCVA	Best-corrected visual acuity
C/D	Cup–disc ratio
CCT	Central corneal thickness
CD	Cell density
EGS	European Glaucoma Society
IOP	Intraocular pressure
ITC	Iridotrabecular contact
LT	Lens thickness
LV	Lens vault
MD	Mean deviation
MIGS	Minimally invasive glaucoma surgery
OCT	Optical coherence tomography
ONH	Optic nerve head
PAC	Primary angle closure
PACD	Primary angle-closure disease
PACG	Primary angle-closure glaucoma
PACS	Primary angle-closure suspect
PAS	Peripheral anterior synechiae
POAG	Primary open angle glaucoma
RNFL	Retinal nerve fiber layer
SD	Standard deviation
SS	Scleral spur
TISA	Trabecular-iris space area
TISA 500	Trabecular-iris space area measured at 500 µm
TISA 750	Trabecular-iris space area measured at 750 µm
